# Spinal Cord Injury as a Socially Lived Disability: A Phenomenological Study of Rehabilitation and Everyday Life Among Community-Dwelling Individuals

**DOI:** 10.3390/jcm15082878

**Published:** 2026-04-10

**Authors:** Dimitra Karadimitri, Christina-Anastasia Rapidi, Stelios Parissopoulos, Dimitrios Skempes, Savvas Spanos, Maria Tsekoura, Vasiliki Sakellari

**Affiliations:** 1 Department of Physiotherapy, School of Health and Care Sciences, University of West Attica, 12243 Athens, Greece; dkaradimitri@uniwa.gr; 2 Department of Physical & Rehabilitation Medicine, General Hospital “G.Gennimatas”, 11527 Athens, Greece; rapidicha@hotmail.com; 3 Department of Nursing, School of Health and Care Sciences, University of West Attica, 12243 Athens, Greece; spariss@uniwa.gr; 4 Department of Physiotherapy, School of Health Sciences, University of Thessaly, 41500 Larissa, Greece; dskempes@uth.gr (D.S.); sspanos@uth.gr (S.S.); 5 Department of Physiotherapy, School of Health Rehabilitation Sciences, University of Patras, 26504 Patras, Greece; mariatsekoura@upatras.gr

**Keywords:** spinal cord injury, paraplegia, neuromuscular impairment, musculoskeletal complications, neurological rehabilitation, community-based rehabilitation, independent living, social model of disability, accessibility, quality of life

## Abstract

**Background/Objectives**: Spinal cord injury (SCI) leads to long-term changes in mobility, bodily function, and everyday participation, extending beyond physical impairment to affect autonomy, identity, and social inclusion. In Greece, limited community-based rehabilitation services, environmental inaccessibility, and fragmented follow-up care further shape the lived experience of individuals with SCI. This study aimed to explore the lived experiences and perceived rehabilitation needs of people with paraplegia living in the community, adopting a phenomenological approach to understand rehabilitation as an ongoing process of reclaiming autonomy, dignity, and participation. **Methods**: A qualitative phenomenological design was employed. In-depth semi-structured interviews were conducted with fourteen individuals with paraplegia following SCI. Data were analyzed using Braun and Clarke’s reflexive thematic analysis, supported by ATLAS.ti software. **Results**: Participants described living with SCI as a *‘Socially lived disability*: *a daily confrontation with an inadequate system and the ongoing struggle for accessibility, autonomy, and dignity’* (Overarching Theme). Participants’ experiences were organized into six themes: (A) facing the new reality, (B) barriers and facilitators of independent living, (C) role and importance of rehabilitation, (D) me and others around me, my difference, (E) the need for adequately trained and informed health professionals and caregivers, (F) ageing as an additional challenge. **Conclusions**: Living with SCI is experienced as an ongoing process of embodied and social reorientation, in which autonomy, participation, and dignity are continuously negotiated rather than restored once and for all. Rehabilitation emerges as a lifelong, person-centered process that extends beyond functional recovery to support bodily confidence, accessibility, social inclusion, and quality of life across the life course. These findings highlight the need for coordinated, community-based rehabilitation systems, accessible environments, and adequately trained health professionals capable of addressing the evolving functional, social, and existential realities of individuals living with SCI.

## 1. Introduction and Background

Spinal cord injury (SCI) is one of the most serious and unpredictable conditions in the nervous system, with multifaceted and long-lasting consequences that extend far beyond physical impairment. SCI can bring about changes on the physical, psychological, and social levels [1]. Individuals living with SCI often experience significant disruptions in their personal identity, autonomy, and social participation, as they adapt to a world largely designed without their inclusion in mind [2]. Rehabilitation spans acute, inpatient, and community phases, with the latter playing a pivotal role in long-term outcomes, social reintegration, and autonomy [3]. Although improvements in acute medical care and inpatient rehabilitation have increased the survival and functional outcomes, the transition to life in the community frequently presents a complex and challenging process marked by both structural and psychosocial barriers.

Rehabilitation is a critical component of long-term adaptation and recovery. The main goal of rehabilitation is to maximize independence in all aspects of life and reintegrate into a productive and fulfilling life [4]. In high-income countries, community-based rehabilitation models aim to support individuals with SCI beyond the hospital setting. However, such systems remain largely underdeveloped or fragmented in many parts of Europe, including Greece [5]. Limited access to services, lack of environmental accessibility, and insufficiently trained professionals often result in increased social isolation, emotional distress, and loss of dignity for individuals with SCI [5,6]. Moreover, spatial inequalities in infrastructure and access to essential services further marginalize people with disabilities [7]. These systemic gaps perpetuate social exclusion and dependency, undermining the goals of rehabilitation itself.

Beyond biomedical perspectives, disability is increasingly understood as shaped by environmental, institutional, and attitudinal barriers that constrain participation and agency [8]. Within this framework, rehabilitation extends beyond functional recovery to encompass autonomy, dignity, and social inclusion [3]. Recent qualitative work also highlights how dominant cultural narratives—such as those perpetuated by the media—reinforce stigmatizing or idealized views of SCI, further shaping how individuals experience their condition [9].

Despite increasing recognition of these broader dimensions, the literature on SCI rehabilitation remains disproportionately focused on clinical outcomes, often neglecting the lived experiences of people as they navigate daily life in the community. Qualitative research provides the appropriate framework for highlighting subjective experiences, personal narratives, and the dynamic relationship of the individual with their environment. In Greece specifically, there is a scarcity of qualitative research focused on community-dwelling individuals’ experiences, particularly regarding access, independent living, and the psychological impact of systemic neglect [5,10]. This gap limits understanding of how rehabilitation is experienced in everyday life and how systemic conditions shape long-term adaptation.

Existing qualitative studies primarily examine in-hospital rehabilitation, including goal-setting, decision-making processes, and experiences with specific physiotherapy interventions [11,12,13,14,15]. In contrast, there is limited evidence capturing the everyday lived experiences of individuals with paraplegia residing in the community, particularly regarding how rehabilitation is understood, accessed, and sustained over time. This represents a critical gap in the literature.

This study addresses this gap by exploring the lived experiences of people with paraplegia living in the community through a phenomenological lens. It seeks to examine how rehabilitation is experienced and embodied as a continuous, person-centered process shaped by environmental, relational, and institutional conditions, and how individuals with SCI navigate everyday life in the ongoing pursuit of autonomy, dignity, and social participation. Within this context, the study aims to illuminate the perceived role of physiotherapy across the life course, moving beyond time-limited or impairment-focused models of rehabilitation.

Guided by this overarching aim, the study addresses the following research questions: (i) How do individuals with SCI experience and make sense of rehabilitation within their everyday lives? (ii) What factors influence their sense of autonomy, dignity, and participation in the community following injury? (iii) How is physiotherapy perceived in relation to ongoing rehabilitation needs, and what barriers and facilitators shape sustained engagement over time?

## 2. Methods

### 2.1. Study Design

This study employed a qualitative phenomenological design to explore the lived experiences of community-dwelling individuals with paraplegia following SCI. Phenomenology was selected as it is particularly suited to examining how individuals experience bodily change, rehabilitation, and everyday functioning, and how meaning is constructed through embodied engagement with the world [16,17]. Using an interpretive orientation, the study conceptualized rehabilitation not as a discrete clinical phase but as an ongoing, contextual and embodied process shaping autonomy, participation, and identity over time. Data were then analyzed using Braun and Clarke’s (2006) [18] reflexive thematic analysis, which is consistent with our focus on meaning-making. Interview questions were carefully designed to elicit in-depth accounts of these experiences. Although thematic analysis is not inherently a phenomenological method, its interpretive orientation allows it to be applied in studies of lived experiences. The six-phase approach facilitated the identification of patterns of shared meaning while preserving the nuance of individual accounts. In retrospect, the interviews elicited rich narratives on functional adaptation, community reintegration, and the evolving role of physiotherapy in daily life. The study was conducted and reported in accordance with the Standards for Reporting Qualitative Research (SRQR) [19] and aligned with key domains of the Consolidated Criteria for Reporting Qualitative Research (COREQ) checklist [20].

### 2.2. Participants and Inclusion Criteria

The study included 14 community-dwelling individuals with paraplegia resulting from spinal cord injury (SCI), aged between 30 and 74 years (mean age = 52.6 years), with time since injury ranging from 2 to 50 years (mean = 20.4 years). The sample comprised 4 women (28.6%) and 10 men (71.4%). Participants varied in terms of neurological level and severity of injury, cause of injury, employment status, and living arrangements. These characteristics were considered important for capturing a diverse range of experiences and are presented in detail in Table 1. While the study was not designed to compare experiences across these characteristics, they provided important contextual background for interpreting participants’ accounts. Participants were purposively divided into two age groups (18–50 years and >50 years) to facilitate exploration of age-related differences in everyday functioning and rehabilitation needs, based on evidence that biological ageing often occurs earlier in people with SCI [21,22]. This approach ensured diversity in experiences related to ageing; however, age was not treated as a primary analytic comparison. Inclusion criteria were: age ≥ 18 years, medically confirmed diagnosis of paraplegia due to SCI, residence in the community, and injury sustained at least two years prior to participation. Recruitment was conducted through purposive sampling via three sites in Athens, Greece: a national patient advocacy organization (the Greek Paraplegic Association), a public General Hospital of Athens with a rehabilitation center (Georgios Gennimatas), and a private neurorehabilitation center (Filoktitis), allowing for the inclusion of participants with diverse rehabilitation trajectories and community reintegration experiences. Participants were recruited through rehabilitation centers and an advocacy organization in Athens. Therefore, findings are interpreted in the context of individuals engaged with these services, which may not fully reflect the experiences of all individuals with SCI in Greece.

### 2.3. Data Collection

Data were collected between June and August 2024. The sample size was guided by methodological recommendations for qualitative and phenomenological research, where relatively small, purposively selected samples (e.g., approximately 5–25 participants) are considered sufficient to generate rich, meaningful insights [23,24]. An initial sampling range of 12–17 participants was defined. However, consistent with qualitative methodological principles, the final sample size was not fixed a priori but was informed by the concept of information power and the richness and relevance of participants’ accounts in relation to the study aim [25].

Recruitment and data collection proceeded iteratively, with ongoing review and comparison of interview transcripts and emerging patterns. From the eleventh interview onwards, no substantively new insights were identified, a pattern that was maintained through the fourteenth interview. At this point, sufficient depth, diversity, and conceptual clarity had been achieved to address the research questions, and recruitment was therefore concluded.

The final sample of 14 participants was thus determined empirically, reflecting the iterative and interpretive nature of qualitative inquiry.

Data were collected through individual semi-structured interviews lasting approximately 45–60 min. Interviews were conducted face-to-face in private, comfortable, and accessible settings, in accordance with participants’ preferences and mobility needs. A semi-structured interview guide was developed to ensure consistency across interviews while allowing flexibility—through clarification and probing questions—to explore issues raised by participants; it included open-ended questions focusing on experiences of rehabilitation, everyday functioning in the community, perceived barriers and facilitators of autonomy, and strategies used to adapt to physical, social, and environmental challenges. The interview guide is presented in Table 2. All interviews were conducted by the lead researcher, while a second researcher was present in the role of observer and systematically documented non-verbal behaviors, interactional dynamics, and contextual features using a structured observation guide to support reflexive data interpretation.

### 2.4. Data Analysis

Interviews were audio-recorded, transcribed verbatim, and analyzed using reflexive thematic analysis following the six-phase framework proposed by Braun and Clarke [18]. Transcripts were coded line by line to capture meaningful units, which were progressively organized into code categories and refined into overarching themes representing patterned meanings across participants’ accounts. The analysis led to an overarching theme that transcended all six themes. Analysis was iterative, moving between narratives and the dataset and literature as a whole. The development of themes and the coding structure are illustrated in Table 3. ATLAS.ti software (version 25; ATLAS.ti Scientific Software Development GmbH, Berlin, Germany) [26] was used to support systematic organization, retrieval, and comparison of codes and themes across interviews. No AI support was used in the coding and synthesis stage.

### 2.5. Rigour

Rigour was ensured in accordance with established qualitative criteria of credibility, dependability, transferability, and confirmability [27]. Credibility was enhanced through a form of investigator triangulation, understood as the inclusion of multiple researchers in the analytic process to enrich interpretive depth. Data analysis was conducted primarily by the first author, with regular discussions among co-authors experienced in qualitative research to critically reflect on emerging interpretations. These discussions did not aim to achieve consensus or coding reliability; rather, they supported a reflexive and interpretive engagement with the data. In this way, the involvement of multiple perspectives contributed to the richness and depth of the analysis, consistent with a reflexive thematic analysis approach [28,29]. Dependability was supported through systematic documentation of analytic decisions, forming an audit trail. Transferability was enhanced through rich description of participants’ accounts and contextual conditions. Reflexivity was integral to the research process, as described in detail in Section 2.6.

### 2.6. Researcher Background and Reflexivity

The lead researcher is a licensed physiotherapist with a Master’s degree in Spinal Cord Injury rehabilitation and is currently undertaking doctoral research in the same field. This sustained clinical and academic engagement afforded in-depth contextual familiarity with spinal cord injury rehabilitation, while also necessitating critical reflexive awareness of potential preconceptions. Consistent with the phenomenological orientation of the study and the reflexive thematic analysis framework proposed by Braun and Clarke [18], the researcher maintained an explicit reflexive stance throughout data collection and analysis, acknowledging the interpretative nature of knowledge production.

Prior to commencing analysis, the lead researcher explicitly documented pre-existing professional assumptions—including assumptions regarding the centrality of physical rehabilitation and physiotherapy in the recovery process—in order to surface and critically examine how these might shape interpretive emphases. These assumptions were revisited at each phase of the analytic process to ensure that the thematic interpretations remained anchored in participants’ own accounts rather than in professionally derived expectations.

Reflexive journaling was maintained systematically throughout both data collection and analysis. Journal entries recorded the researcher’s reactions to interview content, emerging interpretive tendencies, and moments of analytical uncertainty or tension. These entries were reviewed periodically to identify patterns in interpretive positioning and to support critical self-examination of the analytic process.

Regular reflexive discussions were held within the research team, comprising researchers with backgrounds in physiotherapy, physical medicine and rehabilitation, nursing, and qualitative methodology. These discussions served as a deliberate peer-checking mechanism, enabling the team to identify instances where the lead researcher’s clinical background may have foregrounded rehabilitation-oriented interpretations at the expense of other dimensions of participants’ experiences. Interpretive differences were discussed openly and resolved collaboratively, ensuring that the final thematic structure reflected the breadth and complexity of participants’ accounts.

### 2.7. Ethical Considerations

The study was approved by the Research Ethics Committee of the University of West Attica, Greece (approval No.: 50914/27-06-2024). Additionally, necessary permissions were granted by all participating institutions prior to data collection. All participants were fully informed about the study’s aims and provided written informed consent. Data anonymity and confidentiality were rigorously maintained throughout the research process. Pseudonyms—selected by the participants—are used throughout the paper to protect participants’ anonymity.

Given the sensitive nature of the topic and the potential emergence of emotional distress, appropriate distress management procedures were in place. Interviews were conducted by experienced physiotherapist capable of recognizing signs of distress, and participants were reminded of their right to pause or terminate the interview at any time without consequence. In cases where participants exhibited significant emotional distress, a predefined referral pathway was followed, including encouragement to seek professional support and, where appropriate, facilitated referral to relevant mental health services and support organizations.

## 3. Findings

Thematic analysis generated six interrelated themes, each supported by underlying code groups—categories, capturing the complex, multidimensional, and socially situated experiences of living with SCI in Greece (Table 4). Together, these themes are unified by an overarching interpretative theme that conceptualizes SCI as a *Socially Lived Disability*, shaped by ongoing confrontation with systemic inadequacies and a continuous struggle for accessibility, autonomy, and dignity in everyday life. The findings that follow are presented under six thematic sections, each introduced by an analytic overview and illustrated through participants’ accounts. Within each theme, the associated code groups are used to structure and deepen interpretation, highlighting how rehabilitation, everyday functioning, and social participation are experienced and negotiated across different stages of life with SCI. To enhance transparency, frequency-related terms (e.g., “many,” “most,” “several”) are used with an indicative meaning. All six themes were therefore developed based on their relevance to the research question and the richness of the data supporting them, rather than their numerical prevalence alone. Some of them were particularly well-grounded in the data, either because they recurred across multiple participants’ narratives or because they were expressed in depth within individual accounts, sometimes at multiple points within the same interview. This notion of groundedness reflects both breadth (across participants) and depth (within narratives) of our analysis.

### 3.1. Theme A: Facing the New Reality: From Initial Shock to Life Reorganization

The first theme captures the early psycho-emotional and existential experience of the participants following SCI. Initial shock, profound loss, and mourning for pre-injury life dominate this phase. Participants’ narratives illustrate a painful transition from trauma toward the gradual reconstruction of identity, meaning, and life continuity.

#### 3.1.1. Code Category A.1: Loss and Initial Shock

Participants vividly described the existential shock and profound sense of loss immediately following their injury. As Activist (pseudonym) reflected:

*“Once you get over the initial shock of the injury, come out of rehabilitation begin to reintegrate socially there are, of course, difficulties, but over time, you stop thinking about them.”*

*Activist*

Many participants reported disbelief, emotional numbness, and an existential impasse, highlighting the rupture between their past identity and an uncertain future. As Partner explained:

*“When an accident happens to someone, their life changes, and they find themselves at an impasse, questioning who they were and who they are.”*

*Partner*

This existential impasse, marked by delayed meaning-making, was described as an emotionally burdensome process. Similarly, Biker, emphasized the persistent comparison between the pre- and post-injury self, stating:

*“Because there’s always a comparison between how you were before and what you’re trying to do now.”*

*Biker*

Most of participants described the struggle to adapt to a radically altered way of life. Evanthia noted:

*“You find yourself, from one moment to the next, in a completely different situation… It’s difficult to adapt.”*

*Evanthia*

This experience was echoed by Active Man, who described the early period as “*being reborn*”, learning to navigate life anew with a different body.

Overall, this phase was characterized by deep disorientation as participants attempted to come to terms with a changed body, altered identity, and an uncertain future.

#### 3.1.2. Code Category A.2: Emotions: Inadequacy, Grief, and Fear

Feelings of inadequacy, grief and fear permeated the early post-injury period. Participants described mourning not only physical abilities but also lost roles, opportunities and imagined life trajectories. Fear of dependence, social isolation, and future uncertainty was particularly salient. Evanthia articulated the emotional intensity of this phase *“You want to die. You want to see no one. […] You have fears about what the future will hold.”* At another point, she openly acknowledged suicidal ideation, describing a state of profound despair. Fear was also expressed as fear of survival and fear of failure, as she noted: *“At first, I was afraid I wouldn’t make it.”*

The persistent sense of inadequacy was repeatedly emphasized. Maria described the ongoing psychological burden of limitation. She further highlighted the painful social comparison with able-bodied individuals, reinforcing feelings of dependence and exclusion.

*“Living is not easy, it’s not easy, it’s difficult. It’s very difficult. When you see everyone else doing well and walking, while you’re in a wheelchair and help to go anywhere. It’s difficult, it’s very hard.”*

*Maria*

Biker summarized his grief as a difficult psychological adaptation, noting that while he had learned to manage daily life, emotional satisfaction remained elusive. Anger and frustration were also reported, particularly in relation to bodily functions. He stated:

*“I would say that before, I was, let’s say, normal in terms of movement, so this is very difficult. You can’t easily adapt from your previous situation to the new one. That’s the hardest part. It’s more about psychological strength, about how you manage it. I think I’ve reached a level in everyday life where I can manage it. But I’m not happy, I’m not enjoying myself, I can’t do what I want. I’m just adapting.”*

*Biker*

Bill described periods of anger linked to functional limitations and lack of environmental familiarity:

*“Okay, sometimes… okay. There’s also this feeling of, ‘Oh my God, I can’t do the things I used to. Especially in the early days, the first decade or so, when my body hadn’t yet recovered, I had urinary incontinence, and difficulties and questions in the sexual area. I hadn’t learned to navigate places—how to get to the tax office, how get to the hospital. All of that was challenging. There were periods when I felt angry and upset.”*

*Bill*

On the other hand, Taboo Breaker emphasized the irritation caused by daily mobility barriers:

*“The first one to three years are a bit difficult. You get more irritated when you realize that you’re having trouble—let’s say with parking or moving around—and you think that everything used to be fine. Suddenly, you have to struggle just to manage daily life.”*

*Taboo Breaker*

Athlete poignantly concluded: *“Having a spinal cord injury feels like a deprivation of freedom, like being chained or tied down.”*

### 3.2. Theme B: Barriers and Facilitators of Independent Living: The Daily Negotiation of Autonomy

The second theme foregrounds the structural, environmental, and practical challenges encountered by people with paraplegia, alongside the facilitating the factors that enable autonomy and independent living. Issues of accessibility, self-care, and environmental adaptation emerged as central to participants’ efforts to construct a functional and dignified daily life.

#### 3.2.1. Code Category B.1: Lack of Accessibility as an Embodied Experience of Disability

A recurring barrier was the pervasive lack of environmental accessibility, experienced not only as a physical limitation but as an embodied reminder of societal exclusion. Participants described inaccessible public spaces, transportation systems, and buildings as daily tests of resilience and autonomy.

Coucou highlighted the restrictive impact of inaccessible facilities, stating: *“If there isn’t an accessible toilet for me, I simply don’t go.”* Similarly, Basketball Player described navigating Greek urban environments as an ongoing struggle, emphasizing structural neglect and environmental hazards.

Taboo Breaker explicitly framed accessibility as a social and political issue, rejecting the notion that disability is inherent to the body:

*“Shouldn’t I want to do it? Or won’t I be given the chance because the state has decided I will be disabled due to lack of access?”*

*Taboo Breaker*

This perspective was widely shared among participants, who reported a sense of invisibility and devaluation of their needs.

In defining the boundaries of accessibility, Basketball Player succinctly stated: *“Access ends where you can’t go alone.”* Evanthia further linked accessibility to autonomy and social reintegration, emphasizing that without access, social participation and vocational rehabilitation become unattainable.

Overall, the lack of accessibility reinforced feelings of marginalization, dependence, and exclusion, confirming that disability was experienced as socially produced and environmentally enforced, rather than solely medically determined.

#### 3.2.2. Code Category B.2: Assistive Devices and Adaptations: Accessibility Tools

Conversely, assistive technologies and environmental adaptations were identified as key enablers of autonomy and independent living, functioning as facilitators that mitigate embodied barriers to accessibility. Participants emphasized the value of assistive technologies (e.g., wheelchairs) and home modifications, which were perceived as enhancing mobility, safety, and personal control over daily life. However, access to such tools was frequently constrained by financial, administrative, or systemic barriers, limiting their availability and appropriate use.

As Evanthia shared:

*“I have also made the necessary adjustments—that is I’ve set things up so that I don’t need anyone to upload or download anything for me.”*

*Evanthia*

And she continued: *“If you make the appropriate adjustments, you gain extra autonomy and I think it’s best not to depend on anyone.”* These narratives underscore the perception of environmental and ergonomic adaptations as mechanisms through which dependence can be reduced and autonomy enhanced.

At the same time, participants critically reflected on the inappropriate or excessive use of assistive aids, highlighting the importance of need-based and context-sensitive adaptation. Taboo Breaker noted:

*“Don’t build your own little palace of aids with things that you might not actually need for everyday life. You just say they’re convenient, or you learn to use them without stopping to think. For example, there are handles in toilets meant for people with disabilities—but there is no real connection between wheelchairs and people with disabilities. For us, that is a huge difficulty, an obstacle—especially if we can’t stand up or move.”*

*Taboo Breaker*

This perspective illustrates how poorly designed or misapplied accessibility measures may reproduce barriers rather than alleviate them, reinforcing the embodied experience of disability.

Special modifications to vehicles, such as manual-controlled accelerators and brakes, swivel seats, and automated access systems, were described as critical facilitators of mobility-related autonomy. These adaptations enabled participants with paraplegia to drive independently, thereby enhancing professional and social participation and quality of life. As Athlete stated:

*“I got a car with a modified accelerator and brake so I could drive. This changed my life significantly because I became completely autonomous.”*

*Athlete*

This account aligns with the coding of mobility-related autonomy within the broader theme of Barriers and Facilitators of Independent Living.

Furthermore, participants emphasized that the appropriate selection of a wheelchair is crucial for comfort, autonomy, and health-related quality of life. An ergonomic and individually customized wheelchair was described as facilitating daily activities, reducing physical strain, and supporting bodily alignment, thereby enhancing functional independence and self-confidence. As Bill mentioned:

*“But also, from a health perspective—when you sit in a wheelchair that doesn’t fit you, your body is forced into a position that deviates from what is optimal.”*

*Bill*

He further noted: *“I broke my shin not because I wasn’t sitting in a good wheelchair, but because it wasn’t the right wheelchair for me.”* These reflections highlight the interrelation between assistive technology, bodily integrity, and long-term health outcomes.

#### 3.2.3. Code Category B.3: Self-Care and Autonomy: Regaining Daily Skills

Many participants emphasized the importance of relearning self-care routines as a means of reclaiming personal autonomy and functional self-efficacy. Activities of daily living—such as dressing, cooking, personal hygiene, and transfers—were described as critical milestones in the process of regaining independence and as sources of personal pride and self-worth. As Activist explained:

*“Rehabilitation is about teaching you how to function with your existing condition… How to move from a wheelchair to a bed… How to use the toilet, the bathtub, the shower… If you don’t learn to do these things… you end up doing far fewer things than you are capable of.”*

*Activist*

This narrative reflects the coding of retraining in activities of daily living, situating self-care as a core component of personalized and holistic rehabilitation.

Moreover, educational and skills-based programs were highlighted as essential for restoring autonomy beyond basic physical rehabilitation. Activist further emphasized:

*“An educational program that focuses on practical skills… It teaches you how to get out of home* *… There’s a technique. You have to be shown it, trained to do it, master it.”*

*Activist*

These accounts reinforce the distinction between passive rehabilitation and active, participatory learning, positioning self-care as an embodied practice of autonomy rather than a mere functional outcome.

#### 3.2.4. Code Category B.4: Daily Struggle and Practical Difficulties

Despite the presence of assistive devices and acquired skills, independent living was consistently described as a dynamic and continuously negotiated process, characterized by ongoing practical difficulties. Participants reported challenges related to transportation, bureaucratic procedures, service access, and systemic inefficiencies, which contributed to physical fatigue and emotional exhaustion.

When Athlete was asked whether adaptation was more difficult at a practical or psychological level, he responded: *“Both. It turned out to be more difficult in practice, because over time, mental pain eases.”* A similar view was expressed by Biker, who stated: *“I think it was a combination, but more practical.”* These narratives emphasize that practical barriers persist over time, even as emotional distress may gradually subside.

The enduring nature of this struggle was further articulated by Motocross, who described daily life as a continuous effort: *“We are in a constant battle… We have to keep going for this goal. It’s just that the goal keeps changing.”* This metaphor reflects the persistent negotiation of autonomy within constraining environments, aligning with the code category of Daily Struggle and Practical Difficulties.

### 3.3. Theme C: Role and Importance of Rehabilitation: From Treatment to Participation and Inclusion

Rehabilitation was described as a multidimensional, evolving, and meaning-laden process, encompassing both functional and psychosocial dimensions, rather than a time-limited or purely biomedical intervention. Participants emphasized the need for personalized, ongoing, and holistic rehabilitation, highlighting the importance of active engagement through physiotherapy, sport, peer-based support, and the pursuit of personally meaningful rehabilitation goals.

#### 3.3.1. Code Category C.1: Personalized and Holistic Rehabilitation

Activist emphasized the holistic and functional orientation of rehabilitation, distinguishing it clearly from narrowly defined physical treatment:

*“When we talk about rehabilitation, we don’t mean physical rehabilitation. Physical rehabilitation is the least important part. Rehabilitation is about teaching you to function with your existing condition… How will you move from a wheelchair to a bed… How will you use the toilet, the bathtub, the shower… If you don’t learn to do these things… you’ll always need help… and you’ll never be independent.”*

*Activist*

This narrative reflects the coding of retraining in activities of daily living, situating rehabilitation as a process of restoring functional self-efficacy and autonomy.

Similarly, Basketball Player described rehabilitation as an intensive, structured, and participatory daily routine, combining physiotherapy, group exercise, aquatic therapy, self-care training, and gym-based activity: *“At that time, especially at the rehabilitation center… all day, every day, this thing.”* This account illustrates rehabilitation as a comprehensive and immersive process, aligning with the code category of Personalized and Holistic Rehabilitation.

#### 3.3.2. Code Category C.2: Physiotherapy in the Initial Stage and as a Continuous Process

Participants described divergent patterns of engagement with physiotherapy over time. Some reported an absence of systematic or continuous physiotherapy, engaging only in response to acute problems or injuries. As Athlete stated: *“I don’t do anything systematically.”*

Similarly, Evanthia noted: *“I only did physiotherapy when I had a problem with my neck”*, while Activist described episodic physiotherapy related to specific musculoskeletal conditions.

Despite this intermittent engagement, participants consistently acknowledged the importance of physiotherapy as a maintenance strategy, particularly in the absence of other daily physical activities. As Evanthia emphasized: *“I strongly believe in maintenance through physiotherapy… and I consider it essential.”*

In contrast, other participants described ongoing physiotherapy as an integral part of daily life. Biker stated: *“I continue physiotherapy to this day, though over the past year at a less intensive pace”*, while Motocross reported: *“I go out every day because I attend physical therapy.”* These accounts align with the coding of physiotherapy as maintenance and underscore its role as both a source of physical empowerment and a framework for sustaining bodily confidence and functional self-efficacy.

#### 3.3.3. Code Category C.3: Sport as a Means of Reconnecting with the Social Fabric

Participants frequently highlighted sport as a primary avenue for social interaction, inclusion, and peer-based learning. As Taboo Breaker explained, sport facilitates not only physical benefits but also socialization and experiential exposure to alternative ways of living with disability:

*“Exercise generally helps… socializing… meeting other people and seeing what it’s like to interact with this—realizing that this version of life also exists.”*

*Taboo Breaker*

Bill articulated the dual role of sport, prioritizing physical strengthening while emphasizing peer-based sociability and informal knowledge exchange:

*“Mainly muscle strengthening, and sociability second… you also get information… what makes a good wheelchair, who is a good doctor… and other types of information like that.”*

*Bill*

This reflects the coding of peer-based sociability within the code category Sport as a Means of Reconnecting with the Social Fabric.

Additionally, Athlete emphasized that sport enhances functional capacity in activities of daily living, improving transfers, mobility, and overall bodily control. These accounts position sport as a bridge between physical conditioning, functional autonomy, and social participation.

#### 3.3.4. Code Category C.4: Active Rehabilitation: Peer Education and Participatory Learning

Active rehabilitation approaches—particularly, peer education and participatory learning—were described as central mechanisms for empowerment, autonomy, and social reintegration. Peer education was understood as the transfer of experiential knowledge from individuals with lived experience of paraplegia, fostering identification, trust, and psychological empowerment.

Participants stressed the importance of independent living training programs led by peers. Basketball Player stated: “*The people I met through sports… were able to give me the knowledge to avoid mistakes…they protected me.*” He further contrasted peer knowledge with professional expertise, emphasizing the unique experiential authority of peers.

Similarly, Activist noted: *“It is very important to see people who have the same condition as you… so that you can follow their example.”*

For Bill, participation in an active rehabilitation camp represented a turning point in adaptation: *“The active rehabilitation training program was a pivotal point in my adaptation.”* He later characterized its impact as transformative, urging others to participate and highlighting the absence of such programs in Greece.

#### 3.3.5. Code Category C.5: Functional Benefits

Rehabilitation was consistently associated with improvements in mobility, strength, functional independence, and overall quality of life. Motocross reflected: *“As I grow and develop in all aspects, my daily life evolves as well.”*

Active Man described rehabilitation as enabling progressive functional gains necessary for basic activities such as transfers and self-care.

Partner emphasized the importance of professional guidance in physiotherapy to prevent secondary injury and adapt exercise safely following SCI.

Biker highlighted the time-dependent nature of functional gains, describing early rehabilitation as crucial for recovery and later physiotherapy as essential for maintaining achieved functional levels. These narratives align with the coding of improvement in daily functioning and physiotherapy as maintenance.

While many participants highlighted the significance of rehabilitation for functional recovery and autonomy, a few noted limitations or frustrations that contrasted with the dominant experiences, illustrating differing perceptions of rehabilitation services.

#### 3.3.6. Code Category C.6: Psychosocial Benefits

Beyond physical outcomes, rehabilitation contributed significantly to psychological well-being, emotional adjustment, and social integration. Coucou associated physiotherapy with increased energy and improved mood, while Motocross described rehabilitation as facilitating social interaction and engagement with others.

These accounts support the code category of Psychosocial Benefits, underscoring the interdependence of functional and emotional dimensions of rehabilitation.

#### 3.3.7. Code Category C.7: Personal Rehabilitation Goals: “I Want Accessibility”

Across all participants, accessibility emerged as a shared and overarching rehabilitation goal, conceptualized as a prerequisite for autonomy, social participation, and vocational reintegration. As Evanthia stated: *“Access is the key to autonomy and socialization… Accessibility is a chain.”*

This perspective was succinctly captured by Basketball Player: *“Access ends where you can’t go alone.”*

Rehabilitation was thus understood as a means of overcoming accessibility barriers, enabling individuals to pursue desired life activities without environmental constraints.

### 3.4. Theme D: Me and Others Around Me, My Difference: Identity, Diversity, and Relationships

The fourth theme explores the intrapersonal and interpersonal dimensions of the lived experience of paraplegia, focusing on identity construction, experiences of difference and stigmatization, and the dynamics of interpersonal relationships. Participants highlighted how dependency on caregivers, exposure to the social gaze, and perceived differentiation from non-disabled individuals shape both self-perception and social interaction.

#### 3.4.1. Code Category D.1: Experience of Diversity, Stigma, and Disability

A recurring experience described by participants was the confrontation with the social gaze and the sudden awareness of visible differences. As Basketball Player stated:

*“At first, it felt like a huge shock. Walking around and having everyone stare at me.”*

*Basketball Player*

This experience reflects the coding of social gaze and stigma, highlighting the immediate impact of public visibility on self-awareness.

Jason further articulated the contrast between protected rehabilitation environments and broader social spaces, stating:

*“So, when you are in a space where there is understanding and support like a rehabilitation center- even if it’s not verbal, everything feels normal- and then you go out into the world and realize the enormous distance between yourself and other people.”*

*Jason*

This narrative illustrates the transition from an environment of implicit acceptance to one of social differentiation, reinforcing the experience of disability as a socially mediated condition.

Several participants engaged in comparative reflections with non-disabled individuals, emphasizing perceived differences and limitations. As Maria reported:

*“When you see everyone else doing well and walking, and you’re in a wheelchair needing someone’s help just to go wherever you want. It’s hard, really hard.”*

*Maria*

She further elaborated on the effort required to maintain daily functioning, noting:

*“I do all the work. I cook everything, I wash everything, I do everything, but okay, you can’t do it the way a person without a disability does. It’s difficult.”*

*Maria*

These accounts align with the subtheme of experience of diversity and disability, framing difference as an embodied and relational experience.

Athlete contextualized stigma within a broader societal lack of disability education, stating:

*“Experience in these families is something you acquire because no one comes with this kind of education. From school years onwards, in our society, we are generally a bit behind.”*

*Athlete*

This perspective situates stigmatization within structural and cultural limitations, rather than individual attitudes alone.

The majority of participants described changes in social relationships and feelings of difference, yet some expressed experiences of acceptance or resilience that diverged from the prevailing patterns, highlighting the range of social and personal responses.

#### 3.4.2. Code Category D.2: Relationship of Trust and Dependence on Caregivers

The majority of participants reported being largely autonomous in activities of daily living, while acknowledging the availability of support from family members when needed. Biker, who is able to walk short distances with crutches, explained:

*“The doctors advised me to always have someone with me for safety reasons, because it’s easy to fall when using crutches and face various difficulties.”*

*Biker*

This reflects the coexistence of functional independence and precautionary dependence.

Two participants, Evanthia and Jason, reported employing paid caregivers to assist with specific tasks such as *“housework, household cleaning”* and *“shopping at the supermarket”*, corresponding to the coding of use of paid assistance.

Athlete emphasized the importance of caregiver familiarity and experiential knowledge, stating:

*“They have learned and understand- in other words, they know my daily life and the kind of help I might need in order to assist me.”*

*Athlete*

This illustrates caregiving relationships grounded in trust, routine, and mutual understanding.

At the same time, participants stressed the importance of early training in autonomy and self-care to avoid overdependence. As Active Man highlighted:

*“The people around me helped me a lot. But the thing is, when you have people constantly helping you, you don’t adapt, because they do everything for you.”*

*Active Man*

Similarly, Taboo Breaker articulated a strong stance on preserving autonomy, stating:

*“People with paraplegia shouldn’t lose sight of the fact that you’re in a wheelchair, but you have to get used to it and learn how to navigate the world… I don’t accept young people telling me to let their life pass by, have someone constantly behind them or depend on another person.”*

*Taboo Breaker*

These accounts underscore the tension between necessary support and the desire to preserve independence, central to the code category Relationship of Trust and Dependence on Caregivers.

### 3.5. Theme E: The Need for Adequately Trained and Informed Health Professionals and Caregivers: The Importance of Education and Training

This theme reflects participants’ strong emphasis on the role of education, training, and professional competence among health professionals and caregivers. Insufficient knowledge was frequently associated with insecurity, inappropriate practices, and diminished trust in the care system.

#### 3.5.1. Code Category E.1: Education of Caregivers and Therapists

Many participants compared their experiences with rehabilitation teams in Greece and abroad, according to their experiences of hospitalization in rehabilitation centers abroad and in Greece, noting differences in organization, philosophy, and professional training.

As Jason reported:

*“There was a difference between the rehabilitation team in Germany and the one here… in Germany they taught you how to be functional in a wheelchair.”*

*Jason*

This account highlights a functional versus impairment-focused rehabilitation orientation. Activist stated: *“I believe that physiotherapists are specialized, we just don’t have the means in Greece,”* while Biker expressed confidence in professional competence: *“I think they are largely well-trained… At least in the rehabilitation center I attended and in some others I observed. They know their job.”*

Regarding professional qualifications, Partner emphasized the importance of technical expertise and safe handling, noting that inadequate skills could lead to fear and withdrawal from physiotherapy. He further stressed the need for emotional intelligence and therapeutic alliance, stating that professionals should engage with the person holistically rather than focusing solely on physical exercises.

Motocross emphasized the primacy of professional skill and human interaction over technological equipment, asserting that knowledge and commitment outweigh machines.

Finally, Active Man highlighted the importance of lifelong learning, framing professional development as essential for adapting to evolving rehabilitation approaches.

Most participants emphasized gaps in professional knowledge and support, but a few participants shared positive experiences with well-informed professionals, demonstrating that experiences in care provision were not uniform.

#### 3.5.2. Code Category E.2: Ignorance, Lack of Knowledge and Information

Several participants highlighted the lack of caregiver knowledge, particularly within family environments. Coucou described how unawareness of pressure ulcers led to delayed recognition and worsening outcomes.

Similarly, Athlete noted that caregiving knowledge is often acquired through experience rather than formal education, reflecting broader societal gaps in disability-related awareness.

### 3.6. Theme F: Aging as an Additional Challenge: Decreasing Autonomy and Concern for the Future

The final theme brings to the surface the long-standing concerns of the participants as they age with paraplegia. Declining physical ability, fear of losing autonomy, and uncertainty about the future are sources of anxiety and reflection. While participants were purposively recruited across two age groups to ensure diversity of experiences, the analysis did not aim to systematically compare younger and older participants. Instead, age served as a contextual factor that highlighted variation in individual experiences of rehabilitation and community engagement.

#### 3.6.1. Code Category F.1: Gradual Physical Decline

Older participants reported age-related functional decline and increased physical strain. Evanthia stated:

*“Over the years, you lose your strength in general, because I am not young anymore. Back then, I didn’t understand much. It is one thing to be 25 years old and another to be 71. All activity decreases. I believe this happens for most people.”*

*Evanthia*

Partner similarly reflected on bodily deterioration over time, emphasizing the cumulative impact of ageing.

Maria acknowledged reduced mobility: *“I used to walk a lot in one area, and I was very fit. Really fit. Now I don’t walk, I can’t. Years have passed.”* Activist described the accumulation of secondary health conditions, such as joint and shoulder problems.

#### 3.6.2. Code Category F.2: Decreasing Pace of Life

Participants described a slower pace of life and reduced engagement in activities. As Evanthia explained, advancing age was associated with decreased involvement and energy, though certain personal values and persistence remained.

#### 3.6.3. Code Category F.3: Concern for the Future

Most participants expressed uncertainty and apprehension regarding the future, often avoiding long-term planning. Statements from Biker, Coucou, Maria, and Evanthia illustrate a shared tendency to focus on the present moment, reflecting future-related insecurity.

## 4. Phenomenological Synthesis: Essence of the Experience

From a phenomenological perspective, living with spinal cord injury emerges as an ongoing process of existential and embodied reorientation, in which individuals are required to continuously renegotiate their relationship with the body, the self, others, and the world. The experience begins with a profound rupture—an abrupt interruption of bodily continuity, life trajectory, and taken-for-granted normality—accompanied by shock, loss, and grief. This rupture is not confined to the moment of injury but extends into everyday life, shaping how space is navigated, time is perceived, and identity is reconstructed.

As life unfolds after injury, participants’ experiences reveal that autonomy, independence, and participation are not fixed states but fragile achievements, continuously tested through interaction with inaccessible environments, social attitudes, and systemic shortcomings. Disability is thus lived not solely as bodily impairment but as a relational and contextual condition, produced at the intersection of the impaired body, the environment, and institutional arrangements. Everyday activities—such as moving through public space, managing self-care, or engaging socially—become sites of constant negotiation, where autonomy must be actively claimed, defended, and re-established.

Within this landscape, rehabilitation assumes a central existential meaning. It is experienced not as a finite medical intervention but as a lifelong, embodied process of adaptation, empowerment, and meaning-making. Physiotherapy and active rehabilitation enable individuals to rebuild trust in their bodies, regain functional confidence, and reconnect with valued activities, while sport and collective rehabilitation practices foster social repositioning and renewed belonging. Rehabilitation goals gradually shift from restoring bodily function alone to reshaping environmental and social conditions in ways that sustain dignity, accessibility, and life continuity.

Social relationships play a decisive role in this process. Identity is continuously shaped through interaction with others, as experiences of visibility, stigma, support, or misunderstanding influence self-perception and social positioning. Relationships with caregivers and professionals become spaces where dependence and autonomy are simultaneously negotiated, rendering knowledge, competence, and respect essential to lived safety and trust. The presence of informed and sensitive professionals supports autonomy and participation, whereas ignorance amplifies vulnerability and reinforces experiences of marginalization.

Over time, ageing introduces an additional temporal layer to this experience. Ageing with paraplegia intensifies concerns about bodily decline, future dependency, and sustainability of autonomy, making time a salient dimension of lived experience. The past, present, and anticipated future are continuously reinterpreted as individuals seek to preserve meaning, participation, and self-worth within changing bodily and social conditions.

Taken together, the lived experience of spinal cord injury is characterized by continuous striving rather than resolution: a persistent effort to align bodily capacity, environmental conditions, and social relations in order to sustain an autonomous, dignified, and meaningful life. Living with SCI is thus experienced as an enduring struggle—not only against physical limitation, but against systemic inadequacy—requiring resilience, creativity, and ongoing re-engagement with rehabilitation as a foundation for participation and quality of life.

## 5. Discussion

This qualitative study explored the lived experiences of community-dwelling individuals with paraplegia following SCI, recruited through urban rehabilitation and advocacy networks in Athens, Greece, illuminating critical dimensions of everyday functioning, health management, identity, and social participation. The findings demonstrate that life after SCI among this population is shaped through a dynamic and ongoing process of adaptation in which personal, social, environmental, and systemic factors continuously interact. Rather than depicting adjustment as a linear trajectory, the six thematic areas reveal independent living as a negotiated and evolving condition across the life course.

Coping with the “new reality” following SCI emerged as a complex psychosocial and rehabilitative process that extended well beyond physical recovery. Participants described a profound rupture in their anticipated life trajectory, accompanied by the need to reconfigure daily routines, bodily awareness, self-perception, and expectations of function. This disruption is consistent with literature conceptualizing SCI as a form of biographical rupture that requires sustained psychological and embodied adaptation [30]. Importantly, adaptation was not experienced as a finite stage but as a dynamic and fluctuating process marked by ambivalence, loss, resilience, and the ongoing renegotiation of what constitutes functional “normality” in everyday life. From a rehabilitation perspective, these findings highlight the importance of integrating psychological support, therapeutic communication, and patient-centered goal setting within physiotherapy practice across all phases of care, rather than limiting psychosocial input to the acute or inpatient rehabilitation stage. This is supported by recent evidence, such as the randomized controlled trial by Li et al. (2024), which demonstrated that a combined physical-psychological intervention incorporating mindfulness and motivational interviewing improved outcomes for community-dwelling individuals with spinal cord injury [31].

Independent living in the community emerged as a dynamic balance between barriers and facilitators. Persistent structural obstacles—such as limited environmental accessibility and fragmented social services—were mitigated by family support, adaptive strategies, and assistive technologies [32]. Participants described independence not as complete self-sufficiency, but as the ability to exert control, make choices, and engage meaningfully in everyday life. This perspective aligns with social and biopsychosocial models of disability, highlighting that functional limitations arise from interactions between the individual and their environment. Consistent with prior research, among the participants in this study, engagement in community activities and functional outcomes appeared to depend more on accessibility, social support, and assistive devices than on impairment severity alone—findings that resonate with broader evidence in the SCI literature [33,34,35]. Evidence from intervention studies further supports that combining physical activity with psychosocial support can enhance participation, autonomy, and quality of life, underscoring the need to address both personal and environmental factors when promoting independent living.

Rehabilitation emerged as a central pillar not only for functional improvement but also for psychological adaptation, identity reconstruction, and empowerment. Participants emphasized the value of individualized, specialized, and long-term rehabilitation, while simultaneously highlighting discontinuities in care following discharge from inpatient services. The transition from the structured rehabilitation environment to community living represented a critical point of vulnerability for the participants in this study, underscoring the relevance of rehabilitation models that extend beyond institutional settings within similar urban and healthcare contexts. Active rehabilitation programs, in particular, were widely perceived as effective in enhancing functional skills, self-efficacy, social engagement, and overall quality of life—findings that are supported by international evidence [36]. Rehabilitation was thus experienced not merely as physical training but as a process of social repositioning and restoration of agency.

Issues of identity, social relationships, and perceived “difference” were prominent across participants’ narratives. Participants experienced SCI not only as a medical condition but as a socially mediated phenomenon that reshaped how they viewed themselves and how they were perceived by others, aligning with evidence that identity negotiation and reconstruction is a central and complex process following SCI. Qualitative syntheses have described disability identity as multifaceted, involving continuous negotiation between personal self-concept and broader social expectations and roles (identity negotiation and reconstruction following SCI) [37]. Experiences of stigma, social gaze, overprotection, and discomfort within social interactions, including discriminatory attitudes and internalized negative stereotypes, have been linked to diminished self-image, social participation, and psychosocial outcomes in this population [38]. While such dynamics can undermine self-perception and societal engagement, some participants described parallel processes of empowerment and identity reclamation as they adapted meaningfully over time. Collectively, these findings highlight disability as a relational and context-dependent experience, reinforcing the dynamic and negotiated nature of identity following SCI.

A particularly salient finding concerned the need for adequately trained and informed health professionals and caregivers. Participants reported that insufficient specialist knowledge in primary and community care often resulted in fragmented support, preventable complications, and diminished trust in the healthcare system. In contrast, interactions with knowledgeable professionals were characterized by feelings of safety, confidence, and collaborative care. These findings underscore the importance of continuous professional education, interdisciplinary collaboration, and the meaningful involvement of people with SCI in shared decision-making processes.

Ageing emerged as an additional and often under-recognized challenge for the community-dwelling individuals with paraplegia in this study who had been living with SCI over extended periods. Participants described cumulative functional decline, secondary health complications, reduced stamina, and increasing reliance on both formal and informal support, all of which directly affected mobility, endurance, and participation in everyday activities. From a rehabilitation perspective, these findings underscore the need for ongoing, adaptive physiotherapy input rather than episodic or time-limited interventions. The intersection of SCI and ageing introduced new functional and psychosocial vulnerabilities that were frequently inadequately addressed by existing rehabilitation and community services. Consistent with previous research, these findings highlight the importance of long-term rehabilitation planning that views SCI as a lifelong condition requiring periodic reassessment, preventive strategies, and sustained physiotherapy engagement, rather than as a static injury addressed solely in the post-acute phase [30].

Taken together, the findings contribute to existing knowledge by conceptualizing independent living among community-dwelling individuals with paraplegia in an urban Greek context not as a stable achievement but as a continuously negotiated process shaped by accessibility, relationships, rehabilitation, and time. Accessibility, in particular, emerged as an embodied and existential experience rather than a purely technical issue, directly influencing how participants moved, acted, and participated in daily life. The findings align with the social model of disability, while also supporting embodiment perspectives that emphasize the lived and bodily dimensions of environmental interaction. Furthermore, the narratives resonate with life-course and biographical disruption frameworks, highlighting the ongoing renegotiation of identity, autonomy, and participation.

From a methodological standpoint, the analysis was conducted by researchers with professional backgrounds in physiotherapy and rehabilitation, which may have influenced interpretive emphases. To address this, reflexive dialogue was maintained throughout the analytic process, and interpretations were consistently grounded in participants’ own accounts. Although the findings are situated within the Greek sociocultural and healthcare context—characterized by limited accessibility infrastructure and fragmented community services—they are analytically transferable to similar contexts where comparable structural conditions exist.

## 6. Clinical and Practical Implications

While caution is warranted in generalizing beyond the study’s sampling frame, the findings point to the importance of holistic, longitudinal, and participation-oriented care for community-dwelling individuals with paraplegia in comparable urban and healthcare contexts. Rehabilitation professionals, particularly physiotherapists, should extend their focus beyond impairment-based goals to include self-care training, environmental negotiation, caregiver education, and advocacy for accessibility. The strong value attributed to peer support and active rehabilitation suggests that integrating experiential knowledge into formal rehabilitation pathways may enhance autonomy, confidence, and social participation. At the policy level, the results support interventions aimed at improving accessibility, strengthening community-based services, and reducing reliance on informal caregiving structures.

Future studies should adopt longitudinal qualitative designs to examine how autonomy, rehabilitation needs, and social participation evolve over time. Research incorporating the perspectives of caregivers and health professionals would further illuminate relational dynamics influencing independent living, while evaluative studies of peer-led and community-based rehabilitation models could inform the development of more inclusive and sustainable care frameworks.

## 7. Strengths and Limitations of the Study

A key strength of this study lies in its in-depth exploration of lived experience through rich personal narratives, allowing access to meaning-making processes often overlooked in quantitative research. Reflexivity was embedded throughout the research process: the lead researcher’s professional background in neurorehabilitation and physiotherapy informed interpretation, while reflexive journaling and regular team discussions critically examined assumptions and potential biases. Participants were purposively recruited across two age groups to ensure diversity of experiences related to ageing, contributing to a rich and nuanced understanding of autonomy, participation, and rehabilitation across the life course. However, the relatively small sample size and focus on individuals already living in the community may limit the transferability of findings to other populations, such as those in institutional settings. In addition, the study was conducted primarily within an urban context (Athens, Greece), which may not fully capture the experiences of individuals with SCI living in rural or remote areas where access to services and infrastructure may differ significantly. Furthermore, the sample included only individuals with paraplegia, and therefore the findings may not be transferable to those with other forms or severities of SCI, such as tetraplegia. Finally, participants were partly recruited through a national patient advocacy organization (the Greek Paraplegic Association) and two rehabilitation centers (a public rehabilitation hospital and a private neurorehabilitation center), which may introduce a degree of selection bias, as these individuals may be more engaged, informed, or supported compared to those who are less connected to such networks. These limitations should be understood as contextual boundaries rather than methodological weaknesses.

## 8. Conclusions

This study deepens the understanding of community living after SCI among individuals with paraplegia in urban community settings by foregrounding the complexity, variability, and negotiated nature of independence, identity, and participation over time. The findings highlight rehabilitation—and physiotherapy in particular—as a lifelong, adaptive process through which community-dwelling individuals with paraplegia work to sustain functional capacity, autonomy, and meaningful participation within changing social and environmental conditions. Integrating the lived experiences of community-dwelling individuals with paraplegia, such as those who participated in this study, into the design of rehabilitation services, professional training, and policy initiatives is essential for promoting dignity, accessibility, and quality of life across the life course.

## Figures and Tables

**Table 1:** Participants characteristics.

Characteristic	Value
Age, years	Mean 52.6 (range 30–74)
Time since injury, years	Mean 20.4 (range 2–50)
Sex	
Male	10 (71.4%)
Female	4 (28.6%)
Age group	
18–50 years	5 (35.7%)
>50 years	9 (64.3%)
Type of injury	Paraplegia due to spinal cord injury (100%)
Neurological level of injury	T3 (*n* = 1), T4 (*n* = 1), T5 (*n* = 1), T7 (*n* = 1), T9 (*n* = 2), T11 (*n* = 4), T12 (*n* = 2), L1 (*n* = 2)
Completeness	Complete (*n* = 9), incomplete (*n* = 5)
Cause of injury	Traumatic (*n* = 12): traffic accident (*n* = 9), fall (*n* = 3) non-traumatic (*n* = 2): myelopathy (*n* = 1), spinal cancer (*n* = 1)
Living situation	Community-dwelling (100%)
Living arrangement	Alone (*n* = 8), with family (*n* = 6)
Employment status	Retired (*n* = 6), unemployed (*n* = 1), civil servant (*n* = 2), self-employed (*n* = 3), homemaker (*n* = 2)
Educational level	Primary school (*n* = 1) High school (*n* = 3) Associate degree (*n* = 3) Bachelor’s degree (*n* = 4) Master’s degree (*n* = 3)
Recruitment sources	
Greek Paraplegic Association	*n* = 5
General Hospital of Athens “Georgios Gennimatas”	*n* = 6
Filoktitis Rehabilitation Center	*n* = 3

**Table 2:** Interview Guide.

Interview Domain	Indicative Questions
**Introduction & Everyday Context**	What is your life like after the injury? Can you describe a typical day?
**Lived Experience of Rehabilitation**	How have you experienced rehabilitation within your everyday life? How do you make sense of it at this stage?
**Physiotherapy & Ongoing Needs**	What does physiotherapy mean to you? How do you perceive its role in your long-term rehabilitation?
**Goals, Expectations & Outcomes**	What did you hope to achieve through rehabilitation? To what extent were these hopes met? Were there unexpected outcomes?
**Autonomy, Dignity & Participation**	How has the injury influenced your sense of autonomy and dignity? How do you experience participation in your community?
**Barriers & Facilitators to Engagement**	What motivates you to continue—or discontinue—physiotherapy? What obstacles or supports influence your engagement over time?
**Emotional & Existential Responses**	How has this experience affected you emotionally? Have there been moments that significantly shaped how you see yourself or your future?
**Coping & Adaptation**	How have you adapted to life after SCI? What has helped you manage challenges in daily life?
**Social Relationships**	How have your relationships with family, caregivers, or therapists been influenced by your rehabilitation journey?
**Reflection & Closing**	Looking back, would you change anything about your rehabilitation process? Is there something important we have not discussed?

**Table 3:** Coding Table: Examples of Quotes, Codes and Code Categories, Themes.

Participant	Quote	Code	*Subtheme*	Theme
**P3_Partner**	*“When an accident happens to someone, their life changes, and they find themselves at an impasse, questioning who they were and who they are.”*	existential impasse	*Loss and Initial Shock*	**Facing the new reality**
**P2_Evanthia**	*“At first, I was afraid I wouldn’t make it.”*	fear of survival	*Emotions: Inadequacy, Grief, and Fear*	**Facing the new reality**
**P2_Evanthia**	*“The problem is social. When the obstacles are removed, you have no problem afterwards. […] You can move around”*	disability as a social issue	*Lack of Accessibility as an Embodied Experience of Disability*	**Barriers and facilitators of independent living**
**P2_Evanthia**	*“But in general, if you can, today there are many aids available, and you can make the corresponding adjustments. […] If you make the appropriate adjustments, you have extra autonomy and I think it’s best not to need anyone.”*	environmental and ergonomic adaptations	*Assistive Devices and Adaptations: Accessibility tools*	**Barriers and facilitators of independent living**
**P13_Athlete**	*“I got a car with a hand-held accelerator and brake so I could drive. This changed my life a lot because I was completely autonomous.”*	mobility-related autonomy	*Self-Care and Autonomy: Regaining Daily Skills*	**Barriers and facilitators of independent living**
**P1_Maria**	*“Living is not easy, it’s not easy, it’s difficult. It’s very difficult. When you see everyone else doing well and walking, while you’re in a wheelchair and help to go anywhere. It’s difficult, it’s very hard.”*	dependence in daily life	*Daily Struggle and Practical Difficulties*	**Barriers and facilitators of independent living**
**P5_Activist**	*“When we talk about rehabilitation, we don’t mean physical rehabilitation. Physical rehabilitation is the least important part. Rehabilitation is about teaching you to function with your existing condition… How will you move from a wheelchair to a bed.”*	retraining in activities of daily living	*Personalized and holistic rehabilitation*	**The role and importance of rehabilitation**
**P14_Biker**	*“I still use a physiotherapist, I do exercises, I do various things to strengthen and improve, to at least maintain the level I have.”*	physiotherapy as maintenance	*Physiotherapy in the initial stage and as a continuous process*	**The role and importance of rehabilitation**
**P8_Bill**	*“Mainly muscle strengthening, and sociability second. Sociability with peers.”*	peer-based sociability	*Sport as a means of reconnecting with the social fabric*	**The role and importance of rehabilitation**
**P8_Bill**	*“The active rehabilitation camp was a pivotal point in my adaptation.”*	active rehabilitation as turning point	*Active rehabilitation: peer education and participatory learning*	**The role and importance of rehabilitation**
**P13_Athlete**	*“I think that whatever physiotherapy you do is very good for a case like mine. This practice of something for half an hour, an hour, that is, learning something in your body and doing it systematically afterwards has paid off. It helps you, that’s for sure.”*	improvement in daily functioning	*Functional benefits*	**The role and importance of rehabilitation**
**P9_Coucou**	*“As I said, I’ve been doing physical therapy for a long time. The main reason is the pressure ulcers I have. When I used to do it, everything was fine. I was tired, but it was for the best and from what I remember, I also saw a difference in myself. I was much more energetic, more energy compared to now that I don’t do it.”*	psychological enhancement	*Psychosocial benefits*	**The role and importance of rehabilitation**
**P12_Basketball Player**	*“Access ends where you can’t go alone.”*	access as prerequisite for autonomy	*Personal rehabilitation goals: I want accessibility*	**The role and importance of rehabilitation**
**P12_Basketball Player**	*“At first, it felt like a huge shock. Walking around and having everyone stare at me.”*	social gaze and stigma	*Experience of diversity, stigma, disability*	**Me and others around me, my difference**
**P7_Jason**	*“I go to the supermarket with my personal assistant.”*	use of paid assistance	*Relationship of trust and dependence on caregivers*	**Me and others around me, my difference**
**P14_Biker**	*“I believe that a large percentage of them are well-trained.”*	professional training adequacy	*Education of caregivers and therapists*	**The need for adequately trained and informed health professionals and caregivers**
**P9_Coucou**	*“I was sitting for so many hours that I started getting pressure uclers, and because no one in my family knew what they were, we thought they were just a sore. Something happened and they appeared. Over the years, they got worse, and that’s how we learned they were pressure uclers.”*	caregiver lack of knowledge	*Ignorance, lack of knowledge and information*	**The need for adequately trained and informed health professionals and caregivers**
**P2_Evanthia**	*“Over the years. […] You lose your strength in general.”*	age-related decline in strength	*Gradual physical decline*	**Ageing as an additional challenge**
**P2_Evanthia**	*“You generally lose the mood for a lot of activity.”*	reduced activity engagement	*Decreasing pace of life*	**Ageing as an additional challenge**
**P14_Biker**	*“And just like the future, theoretically it is not as bright and auspicious as you get older. Because the difficulties will increase.”*	future-related insecurity	*Concern for the future*	**Ageing as an additional challenge**

**Table 4:** Themes and Code Categories Emerging from the Data.

Overarching Theme	Themes	*Code Categories*	Description/Interpretative Meaning
**Spinal cord injury as a socially lived disability: daily confrontation with an inadequate system and the ongoing struggle for accessibility, autonomy, and dignity.**	Theme A: Facing the new reality	*A1. Loss and Initial Shock*	The experience of loss progressively evolves into a process of adaptation and the search for new life meaning, highlighting the dynamic nature of human resilience.
		*A2. Emotions: Inadequacy, Grief, and Fear*	The initial shock, sadness, and fear are not merely emotional reactions, but meaning-laden experiences that mark the transition from a former “normality” to a new existential condition.
	Theme B: Barriers and facilitators of independent living	*B1. Lack of Accessibility as an Embodied Experience of Disability*	A recurring barrier was the pervasive lack of environmental accessibility, experienced not only as a physical limitation but also as a persistent reminder of social exclusion.
		*B2. Assistive Devices and Adaptations: Accessibility tools*	Assistive technologies and home adaptations were identified as key enablers of autonomy and independent living.
		*B3. Self-Care and Autonomy: Regaining Daily Skills*	Many participants emphasized the importance of relearning self-care routines as a means of reclaiming personal autonomy.
		*B4. Daily Struggle and Practical Difficulties*	Independent living is described as a dynamic and continuously negotiated process, shaped through daily efforts to achieve autonomy.
	Theme C: The role and importance of rehabilitation	*C1. Personalized and holistic rehabilitation*	Rehabilitation is perceived as a multidimensional and evolving process, encompassing both functional and psychosocial dimensions.
		*C2. Physiotherapy in the initial stage and as a continuous process*	Physiotherapy, both during the initial phase and as an ongoing intervention, is experienced as a source of physical empowerment and as a framework for rebuilding bodily confidence and functional self-efficacy.
		*C3. Sport as a means of reconnecting with the social fabric*	Participants frequently highlighted the importance of sport, with many describing it as a primary avenue for social interaction and inclusion.
		*C4. Active rehabilitation: peer education and participatory learning*	Active rehabilitation approaches, including peer education and participatory learning, function as mechanisms of social repositioning, enhancing participation, belonging, and collective meaning-making of disability.
		*C5. Functional benefits*	Rehabilitation supports the restoration of autonomy and physical functioning by enhancing mobility and strength, thereby improving overall quality of life.
		*C6. Psychosocial benefits*	Engagement in rehabilitation contributes to improved psychological well-being and facilitates social integration and emotional adjustment.
		*C7. Personal rehabilitation goals: I want accessibility.*	Accessibility emerged as a central and shared rehabilitation goal across all participants.
	Theme D: Me and others around me, my difference	*D1. Experience of diversity, stigma, disability*	The experience of difference and stigmatization is conceptualized as an existential condition that profoundly shapes self-perception and one’s position within the social world.
		*D2. Relationship of trust and dependence on caregivers*	Relationships with caregivers are structured around mutual trust and dependence, where the need for support coexists with the desire to preserve autonomy.
	Theme E: The need for adequately trained and informed health professionals and caregivers	*E1. Education of caregivers and therapists*	Participants emphasized the critical need for adequately trained health professionals and caregivers.
		*E2. Ignorance, lack of knowledge and information*	Insufficient knowledge and information frequently lead to insecurity, inappropriate practices, and, in some cases, erosion of trust in the care system.
	Theme F: Ageing as an additional challenge	*F1. Gradual physical decline*	Older participants reported increased physical strain associated with age-related functional decline.
		*F2. Decreasing pace of life*	A slower pace of life and heightened vulnerability are accompanied by growing concerns about the future.
		*F3. Concern for the future*	Most participants expressed fear regarding the future, difficulty envisioning long-term plans, and a preference for living in the present.

## Data Availability

The data presented in this study are available on request from the corresponding authors.

## Abbreviations

The following abbreviations are used in this manuscript:

SCI	Spinal Cord Injury
SRQR	Standards for Reporting Qualitative Research
COREQ	Consolidated Criteria for Reporting Qualitative Research
